# The Potential of Metabolomics to Find Proper Biomarkers for Addressing the Neuroprotective Efficacy of Drugs Aimed at Delaying Parkinson’s and Alzheimer’s Disease Progression

**DOI:** 10.3390/cells13151288

**Published:** 2024-07-31

**Authors:** Rafael Franco, Claudia Garrigós, Jaume Lillo, Rafael Rivas-Santisteban

**Affiliations:** 1 Molecular Neurobiology Laboratory, Departament de Bioquimica i Biomedicina Molecular, Universitat de Barcelona, Diagonal 643, 08028 Barcelona, Spain; claudiagarrigos@ub.edu (C.G.); jaumelillo@ub.edu (J.L.); 2 Network Center Neurodegenerative Diseases, CiberNed, Spanish National Health Center Carlos iii, Monforte de Lemos 3, 28029 Madrid, Spain; 3 School of Chemistry, Universitat de Barcelona, Diagonal 645, 08028 Barcelona, Spain; 4 Laboratory of Computational Medicine, Biostatistics Unit, Faculty of Medicine, Autonomous University of Barcelona, Campus Bellaterra, 08193 Barcelona, Spain

**Keywords:** neuroprotection, biomarkers, protein aggregation, neurodegenerative diseases, primary outcome measure, cognition test

## Abstract

The first objective is to highlight the lack of tools to measure whether a given intervention affords neuroprotection in patients with Alzheimer’s or Parkinson’s diseases. A second aim is to present the primary outcome measures used in clinical trials in cohorts of patients with neurodegenerative diseases. The final aim is to discuss whether metabolomics using body fluids may lead to the discovery of biomarkers of neuroprotection. Information on the primary outcome measures in clinical trials related to Alzheimer’s and Parkinson’s disease registered since 2018 was collected. We analysed the type of measures selected to assess efficacy, not in terms of neuroprotection since, as stated in the aims, there is not yet any marker of neuroprotection. Proteomic approaches using plasma or CSF have been proposed. PET could estimate the extent of lesions, but disease progression does not necessarily correlate with a change in tracer uptake. We propose some alternatives based on considering the metabolome. A new opportunity opens with metabolomics because there have been impressive technological advances that allow the detection, among others, of metabolites related to mitochondrial function and mitochondrial structure in serum and/or cerebrospinal fluid; some of the differentially concentrated metabolites can become reliable biomarkers of neuroprotection.

## 1. Introduction

Parkinson’s disease (PD) and Alzheimer’s disease (AD) are the two most common neurodegenerative conditions in developed countries. Due to the increase in life expectancy, the number of patients with PD and AD will increase, mainly because the main risk factor for both diseases is aging. Even in familial PD and AD cases, in which genetic factors are involved, neurodegeneration requires years to be clinically noticeable. In non-familial cases, the underlying causes of neurodegeneration are not fully known, but the clinical symptoms appear late in life.

Unlike diseases affecting peripheral organs, it is not possible to make biopsies of the brain to properly diagnose neurodegenerative diseases. AD is first noticed by cognitive deficits that are shared with other types of dementias. PD is characterized by the appearance (in postmortem samples) of Lewy bodies, which appear in the cytoplasm of affected neurons and are constituted by protein aggregates. Protein aggregates of a presynaptic protein, *α*-synuclein, are found in the neurons of PD patients [1]. PD is considered an *α*-synucleinopathy with Lewy bodies, which are also found in other diseases. The existence of other *α*-synucleinopathies, e.g., multiple systemic atrophy or rapid eye movement sleep behavior disorder without Lewy’s bodies, complicates the diagnosis of *α*-synucleinopathies. Criteria established in 2015 by the Milwaukee (WI, US)-based Movement Disorders Society are used today for PD diagnosis [2]. In parallel, it’s difficult to distinguish Alzheimer’s from other types of dementia [3,4]. In summary, the first diagnosis of PD or AD is not unequivocal.

For decades, the therapy for PD and AD was based on pharmacological approaches. In the case of AD, two types of drugs have been approved. These are acetylcholinesterase inhibitors because increases in acetylcholine in cortical areas of the brain were supposed to have cognitive benefits. Another is based on the allosteric modulation of ionotropic glutamatergic receptors; a lower tone of those receptors could be beneficial in improving cognition and eventually minimizing neuronal death due to glutamate-mediated excitotoxicity. Unfortunately, none of those approaches have led to significant benefits in cognition or in delaying neurodegeneration. In addition, it should be noted that the dozens of natural and synthetic drugs that proved to be efficacious in transgenic mice models of AD have not been approved for AD therapy in humans [5]. More recently, immunological approaches have been pursued, all based on monoclonal antibodies capable of reducing or eliminating amyloid deposits found in the brains of AD patients. The first attempts have failed due to serious side effects and/or a lack of efficacy [6,7], but more antibodies are entering clinical trials to look for efficacy without serious adverse effects. The lack of symptomatic relief from this intervention has sidelined testing other interventions that could be effective in slowing the progression of the disease.

The scenario is better in terms of PD therapy. First of all, several years ago, it was clearly demonstrated that the disease was caused by a deficit of dopamine in certain brain areas and that a physiological precursor able to pass through the blood-brain barrier, levodopa (L-DOPA), if exogenously administered, would markedly reduce the motor symptoms [8,9]. Synthetic compounds able to activate the dopamine receptors expressed in motor control areas have been developed and approved for PD therapy. Dopamine receptor agonists or L-DOPA, which is still used today, must be administered chronically, and this, sooner or later, leads to side effects. The most common side effect is dyskinesia, which consists of abnormal involuntary movements [10]. While there is no good pharmacological approach to limit dyskinesias, deep brain stimulation provides excellent results and is increasingly used [11,12,13,14,15]. As the disease progresses other diverse symptoms can appear and can become serious. Despite the better prospects for the treatment of PD than for the treatment of AD, there is no intervention capable of delaying the progression of the disease.

In recent years, metabolomics has emerged as a powerful tool for understanding the pathophysiology and potential treatment avenues for AD and PD. Metabolomics offers a comprehensive analysis of metabolites, providing insights into the biochemical changes associated with AD and PD and identifying potential biomarkers for early diagnosis and therapeutic targets.

Integrating metabolomics into AD and PD research allows for a deeper understanding of disease mechanisms, enabling the identification of novel metabolic pathways involved in neurodegeneration. By analyzing the metabolic profiles of patients with AD and PD, metabolomics can uncover specific metabolic alterations that may contribute to disease progression and response to treatment.

Introducing metabolomics into the study of AD and PD not only enhances our understanding of these complex diseases but also paves the way for personalized medicine approaches based on individual metabolic profiles. The incorporation of metabolomics in AD and PD research is crucial for advancing our knowledge of disease etiology and for developing more effective, targeted treatments.

This review uniquely emphasizes the critical gap in identifying reliable biomarkers for neuroprotection in Alzheimer’s and Parkinson’s diseases, an aspect that has been underexplored in previous reviews. In contrast to existing reviews, we integrate recent advances in metabolomics and their potential to uncover novel biomarkers in plasma, cerebrospinal fluid, urine, and saliva, providing a comprehensive approach to neuroprotective strategies.

Our review stands out by not only discussing the limitations of current therapeutic approaches but also proposing that innovative metabolomic techniques could bridge the gap between preclinical findings and clinical applications in neuroprotection. Emphasizing the role of metabolomics in AD and PD provides a more holistic view of disease mechanisms and highlights the importance of metabolic pathways in the search for novel therapeutic strategies. In addition, the findings already reported (described in Section 7) should guide clinical trials to include metabolomics parameters in assessing the efficacy of interventions aimed at combating neurodegenerative diseases.

## 2. Recent Data on Neuroprotection Using Animal Models of Parkinson’s and Alzheimer’s Diseases

Table 1 illustrates recent data on the diversity of animal models and therapeutic interventions for PD. Various models, including MPTP-induced lesions in mice, transgenic mice, and *Caenorhabditis elegans*, are used to study the disease and test potential treatments.

Interventions include kurarinone, tauroursodeoxycholic acid (TUDCA), celastrol, curcumin, urolithin A, osmotin, withaferin A, ceftriaxone, Dl-3-n-butylphthalide, and necrosulfonamide. These interventions demonstrate neuroprotective effects by improving motor behavior and reducing neuroinflammation, among other mechanisms. However, while these studies explore various mechanisms of action, such as inhibition of apoptotic pathways, reduction of reactive oxygen species, and attenuation of pro-inflammatory responses, they do not propose specific biomarkers useful for determining the degree of neuroprotection that could be achieved in human patients.

The conclusion derived is that advances in neuroprotection mechanisms in animal models of PD do not translate into the discovery of useful biomarkers for measuring neuroprotection in Parkinsonian patients. It should be noted that these studies make no attempts to identify specific markers in plasma or serum. We think that the most used models, which involve treating rodents with a neurotoxin, are not suitable for metabolomics studies using plasma, urine, etc. Neurotoxin treatment likely results in metabolic changes reflected in the abnormal composition of amino acids, lipids, and other substances in body fluids. These alterations do not correlate with the metabolic changes observed in patients.

Table 2 highlights recent findings on a diverse array of animal models and therapeutic interventions for AD. These models, including transgenic mice and rodents administered ß-amyloid intracerebrally, are employed to study the disease and assess potential treatments.

Interventions include NFPS, norboldine, chlorogenic acid (CGA), butyrylcholinesterase inhibitor UW-MD-95, RG2833, Gas-miR36-5p, TCRAß-Tregs, exercise training combined with postbiotic supplements, isolinderalactone, aucubin, methionine, conifer essential oils, TO901317, anthocyanin-rich blueberry extracts, geniposidic acid and nano-honokiol. These interventions demonstrate neuroprotective effects, often evidenced by improved learning and memory and reduced amyloid-beta (Aß) deposition, among other outcomes.

While these studies explore various mechanisms, such as GlyT1 inhibition, modulation of the AMPK/GSK3ß/Nrf2 pathway, and inhibition of the JNK signaling pathway, they do not propose specific biomarkers useful for determining the degree of neuroprotection that could be achieved in patients.

The conclusion is that advancements in understanding neuroprotection mechanisms in animal models of AD do not translate into the discovery of useful biomarkers for measuring neuroprotection in AD. It should be noted that these studies do not endeavor to identify specific markers in plasma or serum.

## 3. Clinical Trials for Parkinson’s and Alzheimer’s Diseases

The first aim of the paper was to highlight the lack of tools to measure whether a given intervention affords neuroprotection in patients with AD or PD. In other words, is any drug or antibody being tested for efficacy in preventing neurodegeneration? Preclinical research has been instrumental in providing mechanisms of neuroprotection in both animal models of PD and AD. Then, it would be expected that some pharmacological or immunological approach could demonstrate efficacy against neurodegeneration in clinical trials. We have then considered those clinical trials that are registered on the ClinicalTrials.gov site searching for “Alzheimer and dementia” or for “Parkinson’s disease” under the “intervention” field in the https://clinicaltrials.gov (accessed on 6 October 2023). The summary of the data derived from the clinical trials presented below is that neuroprotective biomarkers do not yet exist. It should be noted that biomarkers of neuroprotection could be useful for diagnosis, and, in this sense, the discovery of biomarkers for diagnosis and estimation of the progression of the disease comes before the discovery of biomarkers of neuroprotection. The clinical trials listed in Table 1 and Table 2 mainly rely on test scores and not on biomarkers, confirming again the lack of biomarkers for assessing the efficacy of interventions to combat neurodegenerative diseases.

## 4. Clinical Trials for Parkinson’s Disease with Results

For the search of clinical trials on the https://clinicaltrials.gov/ (accessed on 1 July 2024) database we used “Parkinson’s disease” in the “intervention” search engine, and results were filtered by being in phase 3, i.e., those assessing efficacy. Trials that were both interventional, whose data of completion was between 1 January 2018, and 30 November 2023 and had results were then considered; listed in Table 3.

Despite being posted as phase 3, there are a significant number of trials whose primary outcome addresses safety rather than efficacy. A limited number of studies assess the efficacy of new compounds compared to existing therapies, thus suggesting that the current therapy for symptoms is largely optimized. The tozadenant trials exemplify the difficulty in improving the current pharmacological therapies (two clinical trials with tozadenant are listed in Table 3). The drug has not met expectations, and it would not likely be approved due to adverse effects. It should be noted that, for ethical reasons, any intervention in patients already taking L-DOPA should maintain the medication, meaning that adverse effects may be due to the assayed drug, e.g., tozadenant, or by the tozadenant plus L-DOPA combination.

Some studies attempt to improve the treatment of PD by assuming that a more stable concentration of L-DOPA in plasma would cause fewer side effects. In fact, the oral administration of pills containing L-DOPA leads to a peak of high concentration followed by a sustained decrease that is maintained until the intake of another pill. Patches of rotigotine, a dopaminergic receptor agonist, and powders of L-DOPA to be inhaled were approved for PD therapy several years ago [44,45]. Table 3 lists the trial using CVT-301, a powder that can be self-administered during off-periods, but the outcome measures are related to pulmonary safety concerns. Another clinical trial that tests the efficacy of novel forms of L-DOPA administration includes subcutaneous injections of levodopa and carbidopa. Gels of levodopa/carbidopa administered parenterally are assessed in advanced PD cases, and the outcome measures include the determination of scores for dyskinesia and non-motor manifestations (Table 3).

Finally, some studies aimed at addressing complications derived from PD and/or the therapy. One of the adverse events that may appear in the course of the disease is psychosis, and, therefore, some drugs enter clinical trials to address psychotic symptoms. Neither are biomarkers of biochemical parameters in the blood (or cerebrospinal fluid (CSF)) used as a primary outcome. 

Some studies use unconventional primary outcomes like subject compliance with rules or the number of dropouts. The idea behind this approach is to extrapolate the intervention’s benefits by comparing data from patients who discontinue the therapy with those who do not. A notable example is the NCT03773796 trial evaluating nabilone, a Δ^9^-tetrahydrocannabinol (Δ^9^-THC) analog, which has up to nine primary outcome measures ranging from compliance and postural blood pressure changes to hallucinations and suicidal ideation (Table 3).

There is only one trial on diagnosis listed in Table 3 (NCT04193527). It consists of positron emission tomography imaging (PET) using radiolabeled (iodinated, ^123^I) ioflupane, a cocaine analog. Sensitivity and specificity in the blinded independent analysis are used as primary outcome measures in a cohort consisting of 74 PD patients, 74 patients with essential tremors, and 22 healthy volunteers. Despite PET being a powerful technological development and the use of ^123^I-ioflupane has been approved, the results of the study show that the diagnosis of PD using ^123^I-ioflupane presents uncertainties. Sensitivity and specificity values varied depending on the analyst; sensitivity values provided by the three analysts were: 0.851, 0.865, and 0.919, and specificity values were: 0.933, 0.960, and 0.813. The large difference found between the highest and lowest specificity values is worrying and suggests that the field of PD needs to find appropriate markers for an unequivocal diagnosis and the evaluation of disease progression.

## 5. Clinical Trials for Alzheimer’s Disease with Results

On the search for clinical trials on the https://clinicaltrials.gov/ database (accessed on 1 July 2024) we used “Alzheimer’s disease” in the “intervention” search engine and were filtered by being in phase 3, i.e., those assessing efficacy. Trials that were both interventional, whose data of completion was between 1 January 2018, and 30 November 2023, and had results were then considered; they are listed in Table 4. 

Interventional clinical trials for AD, which are listed in Table 4, are quite different from interventional trials for PD. Whereas PD has efficacious medication to deal with symptoms, neither acetylcholinesterase inhibitors nor modulators of ionotropic glutamate receptors improve the cognitive deficits of AD or delay disease progression. However, in both AD- and PD-related clinical trials, the primary outcome measures consist of test scores, which are more motor-related in PD and more cognition-related in AD. The primary outcome of the first trial listed in Table 4 (Ref. Number: NCT04623242) is “cognitive efficacy”, which is measured by several tests, namely the Mini-Mental State Examination (MMSE), the Wechsler Memory Scale-Revised Logical Memory Delayed Recall (MEMUNITS), the International Shopping List Task (ISLT), and the Wechsler Adult Intelligence Scale Digit Symbol Substitution (WAIS) tests. The goal of the trial is very ambitious as it uses anti-amyloid ß (Aß) antibodies, gantenerumab administered subcutaneously, or solanezumab administered intravenously, to verbatim “prevent dementia” in patients with early-onset familial AD. Table 4 includes nine more clinical trials that use monoclonal anti-amyloid ß antibodies; except in one case, efficacy is measured by different tests and not via biomarkers. The exception is the NCT05108922 trial (see end of section) on PET. It should be noted that the NCT03491150 trial “An Open-Label Crenezumab Study in Participants with Alzheimer’s Disease” considers the presence of anti-crenezumab antibodies as one of the two outcome measures (the first one is the appearance of adverse events). It is intriguing why this was an outcome measure, and it is also intriguing why 76 and 73 individuals on placebo and crenezumab, respectively, were considered for the occurrence of adverse events, while none (either on placebo or crenezumab) for the measurement of anti-crenezumab antibodies.

The NCT03131453 trial uses CNP520 (Umibecestat), a BACE ß-secretase-1 inhibitor, and again, primary outcome measures include a cognitive test score and time to diagnosis (AD-related mild cognitive impairment or AD-related dementia). 

**Table 3:** Clinical trials related to Parkinson’s disease. Selected as being registered on Clinicaltrials.org, and for being interventional and having results. See details in text. Characteristics: 65 years or older; phase 3, interventional with results.

Ref Number	Title	Primary Outcome Measures	Biomarkers	Country and Year of Completion
NCT03051607	Safety and Tolerability of Tozadenant as Adjunctive Therapy in Levodopa-Treated Patients with Parkinson’s Disease.	To Evaluate the Safety and Tolerability of Tozadenant in Levodopa-treated PD Patients Experiencing Motor Fluctuations.	No	US 2018
NCT02453386	Safety and Efficacy Study of Tozadenant to Treat End of Dose Wearing Off in Parkinson’s Patients	Change from Baseline to Week 24 in the Number of Hours Per Day Spent in OFF Time	No	US 2018
NCT02242487	Twelve-Month Safety and Efficacy Study of CVT-301 In Parkinson’s Disease Patients with OFF Episodes	1 Pulmonary Safety of CVT-301 Change from Baseline for FEV1. 2 Pulmonary Safety for CVT-301 Change from Baseline for FVC. 3 Pulmonary Safety for CVT-301 Change from Baseline for (FEV1/FVC).	No	US 2018
NCT03670953	A Study to Evaluate the Safety and Efficacy of IPX203 in Parkinson’s Disease Participants with Motor Fluctuations	Mean Change from Baseline in “Good on” Time Per Day at Week 20/Early Termination (ET)	No	US 2021
NCT03781167	A Study to Evaluate the Safety and Tolerability of ABBV-951 in Subjects with Parkinson’s Disease (PD)	Number of Participants with Adverse Events, among others: death, is life-threatening, requires or prolongs hospitalization, results in a congenital anomaly, persistent or significant disability/incapacity	No	US 2022
NCT04380142	Study Comparing Continuous Subcutaneous Infusion Of ABBV-951 with Oral Carbidopa/Levodopa Tablets For Treatment Of Motor Fluctuations In Adult Participants with Advanced Parkinson’s Disease	Change from Baseline to Week 12 of the Double-Blind Treatment Period in Average Daily Normalized “On” Time without Troublesome Dyskinesia	No	US 2021
NCT02799381	A Study Comparing Efficacy of Levodopa-Carbidopa Intestinal Gel/Carbidopa-Levodopa Enteral Suspension and Optimized Medical Treatment on Dyskinesia in Subjects with Advanced Parkinson’s Disease (DYSCOVER)	Mean Change from Baseline to Week 12 in Unified Dyskinesia Rating Scale (UDysRS) Total Score	No	US 2019
			No	US 2018
NCT02549092	A Study to Examine the Effect of Levodopa-Carbidopa Intestinal Gel (LCIG) Therapy Relative to That of Optimized Medical Treatment (OMT) on Non-motor Symptoms (NMS) Associated with Advanced Parkinson’s Disease	1 Change from Baseline to Week 26 in the NMSS Total Score 2 Change from Baseline to Week 26 in the Modified PDSS-2 Total Score	No	US 2022
NCT00660673	Open Label Continuation Treatment Study with Levodopa-Carbidopa Intestinal Gel in Advanced Parkinson’s Disease	Number of Participants with Treatment-emergent Adverse Events, among other: resulted in death, was life-threatening and required inpatient hospitalization or prolongation of an existing hospitalization	No	US 2021
NCT03329508	A Phase 3 Study with P2B001 in Subjects with Early Parkinson’s	Change in Total Unified Parkinson’s Disease Rating Scale (UPDRS) Score (Defined as Sum of Parts II and III, Scores (0–160).	No	US 2021
NCT00550238	A Study of the Safety and Tolerability of Pimavanserin (ACP-103) in Patients with Parkinson’s Disease Psychosis	Safety: Number (%) of Patients with Drug-related Treatment-emergent Adverse Events (AEs)	No	US 2018
NCT03773796	Nabilone for Non-motor Symptoms in Parkinson’s Disease	1 Adverse events in PD Patients Taking Nabilone, Between Visit (V) 1 and V 3 (6 months) 2 Number of Subjects (%) Who Discontinue the Study Due to an AE Between V 1 and V 3 3 Number of Subjects (%) Who Discontinue the Study Due to Other Reasons Than an AE Between V 1 and V 3 4 Suicidality in PD Patients Taking Nabilone Between V 1 and V 3 Using the Columbia-Suicide Severity Rating Scale 5 Hallucinations in PD Patients Taking Nabilone Between V 1 and V 3 6 Day-time Sleepiness in PD Patients Taking Nabilone Between V 1 and V 3 7 Orthostatic Hypotension in PD Patients Taking Nabilone between V 1 and V 3 8 Subject Compliance in PD Patients Taking Nabilone. 9 Changes in Supine and Standing Blood Pressure Measurements (mmHg) in PD Patients Taking Nabilone Between V 1 and V 3	No	Austria 2020
NCT03391882	A Study of an Investigational Drug to See How it Affects the People with Parkinson’s Disease Complicated by Motor Fluctuations (“OFF” Episodes) Compared to an Approved Drug Used to Treat People with Parkinson’s Disease Complicated by Motor Fluctuations	Change from Pre-dose to 90 Mins. Post-dose in Movement Disorders Society Unified Parkinson’s Disease Rating Scale Part III Score After 4 Weeks of Dosing in Each Crossover Period (Assessed by the Blinded-rater In-clinic at Visit 3 and Visit 6 of PART B).	No	Austria, France 2021
NCT03971617	Clinical Trial to Evaluate the Safety and Tolerability of Hydrogen in Patients with Parkinson’s Disease	Number of Treatment-emergent Adverse Events	No	US 2021
NCT03877510	Open Label Extension (OLE) Study of the Safety and Clinical Utility of IPX203 in Parkinson’s Disease (PD) Participants with Motor Fluctuations	Number of Participants with Treatment-Emergent Adverse Events, defined as “any unfavorable and unintended sign (e.g., an abnormal laboratory finding), symptom, or disease temporally associated with the use of a medicinal product, whether or not considered related to the medicinal product”.	No	US 2022
NCT02202551	Open-Label Safety Study of ADS-5102 in PD Patients with LID	Number of Participants with Reported AEs and Safety-Related Study Drug Discontinuations	No	US 2018
NCT02168842	Efficacy of Isradipine in Early Parkinson Disease	1 Adjusted Mean Change in Total Unified Parkinson Disease Rating Scale (UPDRS) Score 2 Adjusted Mean Change in Adjusted UPDRS Score	No	US 2018
NCT03829657	Phase 3 Clinical Effect Durability of TD-9855 for Treating Symptomatic nOH in Subjects with Primary Autonomic Failure	Proportion of Participants with Treatment Failure at Week 6 of RW Treatment Period	No	US 2021
NCT04193527	A Study to Evaluate the Diagnostic Efficacy of DaTSCAN™ Ioflupane (123I) Injection in Single Photon Emission Computed Tomography (SPECT) for the Diagnosis of Parkinsonian Syndrome (PS) in Chinese Patients	1 Sensitivity Analysis of the Blinded Independent Read of DaTSCAN™ SPECT Images 2 Specificity Analysis of the Blinded Independent Read of DaTSCAN™ SPECT Images	No	China 2021
NCT03325556	Relapse Prevention Study of Pimavanserin in Dementia-related Psychosis	Time from Randomization to Relapse in the Double-blind (DB) Period	No	US 2019
NCT04095793	Phase 3 Open-Label Extension Study of TD-9855 for Treating Symptomatic nOH in Subjects with Primary Autonomic Failure	Number of Participants with Treatment-emergent Adverse Events (TEAEs)	No	US 2021
NCT03750552	Clinical Effect of Ampreloxetine (TD-9855) for Treating Symptomatic nOH in Subjects with Primary Autonomic Failure	Change from Baseline in Orthostatic Hypotension Symptom Assessment (OHSA) Question #1 Score at Week 4	No	US 2021

A vaccination-like approach aimed at producing anti-Aß antibodies involves injecting a chimeric protein consisting of a central amino acid sequence linked to several molecules of the Aß peptide. CAD106 (amilomotide) is a protein designed to produce antibodies against the N-terminal part of Aß-specific without unrolling any Aß-specific T-cell response. Amilomotide consists of multiple copies of the Aß_1-6_ peptide derived from the N-terminal B cell epitope of Aß, coupled to the protein of Qß bacteriophage that assembles into a virus-like particle [46]. This multimeric assembly exhibits immunogenicity, eliciting the production of antibodies against the Aß_1-6_ epitope, thus presenting the potential to reduce amyloid burden. A limited clinical trial in 2012 showed safety and concluded that CAD106 leads to an “acceptable antibody response in patients with Alzheimer’s disease” [47]. The study was promising, but larger trials and additional research were suggested. The NCT03131453 trial uses this approach, in which, again, the primary outcomes include a cognitive test score and time to diagnosis (AD-related mild cognitive impairment or AD-related dementia). The NCT02565511 trial, with similar outcome measures, combines CAD106 with CNP520. According to Alzheimer’s News Today, “sponsors found that patients taking CNP520 showed a decline in some cognitive scores and decided to discontinue that drug’s portion of the trial”; however, the CAD106 branch is ongoing and will likely conclude in March 2025 (https://alzheimersnewstoday.com/cad106/; accessed on 19 November 2023).

Repurposing strategies are attempted, for instance, using AVP-786, which combines quinidine, approved to combat arrhythmias and dextromethorphan, a CNS drug used as a cough reliever. The trials using AVP-786 (NCT02442765 and NCT02442778) also consider test scores as primary outcomes. 

In none of the trials listed in Table 4, biomarkers are used as primary outcome measures, and four of them consider PET data. One of them (NCT05108922) uses the florbetapir PET tracer to assess amyloid burden, comparing the effects of two different monoclonal antibodies. Another (NCT03901092) uses a different tracer, ^18^F-AV-1451, but with the same rationale of measuring reductions in the PET signal in AD-affected brain areas. Florbetapir, or AV-1451 PET imaging, is just one tool among many used in diagnosing AD, and it is often used in conjunction with other clinical assessments and tests. In summary, PET imaging using current tracers can be used to measure plaque reduction but not as an objective measure of neuroprotection. A similar approach, but using ^18^F-flortaucipir, a tracer for Tau, was followed in trials NCT03901105 and NCT02516046. One of the conclusions of the trials is that PET scans (premortem) when using a standardized uptake value in the cortex, can correlate with quantitative measures of pTau in postmortem autopsies. Another conclusion is that the ^18^F-flortaucipir-based PET methodology “may provide valuable information regarding the risk of clinical deterioration over 18 months among patients with AD and mild cognitive impairment” [48,49]. Therefore, to date, these PET protocols can be used to evaluate risks but are not suitable to evaluate neuroprotection.

## 6. Assessment of PD and AD Diagnosis and Disease Progression Based on Proteomics

Given that pathological hallmarks in many neurodegenerative diseases include protein aggregates, proteomics approaches have been attempted for diagnosis and disease. Most studies focused on Aß in the case of AD and on α-synuclein in the case of PD. More recently, Tau and pTau have been considered in the case of AD [48,50,51,52,53,54].

Without entering into the discussion about whether aggregates are a cause or consequence of the diseases [55,56], the main challenge associated with targeting these proteins comes from the inherent biochemical properties of aggregation-prone proteins.

### 6.1. Assessment of AD Diagnosis and Disease Progression Based on Aß or pTau

There is debate about which specific form of Aß can provide useful information in terms of diagnosis and disease progression in AD. When assessing the neurotoxicity of Aß, it is necessary to manipulate the protein to obtain a mix of species that differ in the degree of aggregation [57,58]. This issue does not happen in proteins used for the diagnosis of other diseases, in which the concentration of the protein (for instance, in serum) is the sole parameter that helps to decide any clinical diagnosis or intervention. Is the concentration of monomers of Aß_1-42_ in serum or CSF enough? Should it be higher in AD? Should it increase throughout the progression of the disease? Should the relationship between the concentrations of the monomeric and dimeric forms of Aß be the parameter to be determined? Should it be higher in AD? Should it increase throughout the progression of the disease? [59]. Irrespective of the answer, a second serious inconvenience is the difficulty in distinguishing monomers/dimers/trimers/etc. of proteins in a clinical chemistry laboratory. In other words, clinical chemistry would not incorporate a new parameter unless it was fully clear what was to be measured and where [60].

Until recently, the possibility of using pTau as a biochemical parameter for diagnosis had little hope because of the difficulty in defining which specific form of Tau should be measured. Tau is prone to hyperphosphorylation, and it has been difficult to define which phosphopeptide(s) are essential to aggregation and whether such peptide(s) would reach the serum of the CNS without being dephosphorylated [61]. Basing a diagnostic or prognostic assessment on the ratio of all phosphorylated Tau isoforms to total Tau would likely lack sufficient discriminatory power to be clinically relevant. However, a recent report has shown the usefulness of a commercial kit to determine phosphorylated Tau 217 in plasma to detect Alzheimer’s disease [62]. The enthusiasm aroused by this discovery should be modulated by the fact that the positive correlation shown in the paper was obtained with markers that do not objectively reflect the status of the disease, i.e., it seems as if the pTau 217 is validated using other biomarkers that are not validated themselves [62]. Furthermore, the plasma level of pTau 217, even if instrumental for diagnosis, may not be appropriate for the assessment of neuroprotection.

### 6.2. Assessment of PD Diagnosis and Disease Progression Based on α-Synuclein

Research efforts in Parkinson’s disease have followed a trajectory parallel to those in Alzheimer’s disease, with α-synuclein taking the central role. The current situation is similar in terms of the enormous number of studies that use the determination of α-synuclein in blood plasma and/or CSF to confirm the diagnosis and evaluate the progression of the disease.

A major problem that hinders PD diagnosis is that it is not the only disease that presents α-synuclein aggregates. There exists a spectrum of disorders, collectively termed synucleinopathies, characterized by the cerebral accumulation of α-synuclein aggregates. However, the main issue derives once more from the biochemical properties of sticky proteins, i.e., proteins interact together (and with other proteins) to form aggregates. For routine analysis in a clinical chemistry setting, it would be a challenge to distinguish between monomers/dimers/etc. of α-synuclein. In addition, mutations in the α-synuclein gene, SNCA, would lead to changes that favor dimer/oligomerization in comparison with non-mutated forms. Therefore, the uncertainties are similar to those of Aß/pTau-based biomarkers in AD: Is the concentration of monomers of α-synuclein in serum or in CSF enough for detection? Should it be increased in PD? Should it increase throughout the progression of the disease? Should the relationship between the concentrations of the oligomeric and monomeric forms of α-synuclein be the parameter to be determined? Should it be increased in PD? Should it increase throughout the progression of the disease? An added complication is α-synuclein degradation, with resulting proteolytic products appearing in plasma and serum samples. Technological approaches to measuring α-synuclein in plasma should consider whether to include or discard degradation products when determining the overall α-synuclein concentration.

α-synuclein seed amplification assays, a method to detect the formation of α-synuclein aggregates, apparently have the potential to distinguish PD patients from healthy controls [63]. The method is proposed as capable of detecting patients before the appearance of clinical symptoms and the status of individuals who are at risk due to genetic factors [64]. More studies are needed to confirm these expectations. Difficulties arise from the need to obtain CSF, which may not be obtainable in people without clinical symptoms, and the challenge of standardizing a method to quantitatively measure “seed amplification”.

### 6.3. Assessment of AD or PD Diagnosis Using Other Protein Markers

It is important to emphasize the limitations and challenges involved in translating findings related to putatively neuroprotective proteins from experimental in vitro assays or in vivo animal models of PD or AD to real-world clinical applications or reliable outcome measures in human trials. An example is Nrf-2, a transcription factor known for its role in cellular defense signaling and for participating in the mechanisms underlying the actions of apparently neuroprotective drugs [65]. There are neurotrophic factors and their receptors that are of interest from the point of view of the pathophysiology of PD and AD. Decreased brain levels of neurotrophic factors may be a factor to consider in neurodegenerative diseases, but there is also the opposite possibility, namely, that impaired receptor function may drive increases in the levels of some of those factors [66,67]. In addition, neurotrophic factors may not cross the blood–brain barrier to appear in plasma or may be unstable or rapidly degraded [68,69,70].

Indeed, the translation of laboratory results to clinical use in terms of biomarker of neurodegeneration or neuroprotection is hampered by several factors. What works effectively in a mouse or rat model may not necessarily translate to similar effects in humans. Regarding this, we can highlight the complexity of human systems. Human neurodegenerative diseases are complex and multifactorial. While in vitro and animal models provide invaluable insights, their relative simplicity may not fully recapitulate all factors that influence neurodegenerative disease progression in human patients. Moreover, the variability in human biology, genetics, and environmental factors poses a challenge to finding universal markers that can reliably indicate disease status or treatment efficacy. Factors such as patient heterogeneity, and varieties in disease progression contribute to this difficulty. However, optimism remains that further research can uncover the appropriate avenues for progress in diagnosing and combating AD and PD, aided by continued advances in the field of personalized medicine.

## 7. The Potential of Metabolomics and Discrimination Analysis in AD/PD Diagnosis and Assessment of Disease Progression

It is intriguing why easy-to-obtain clinical chemistry parameters are not used more often in clinical trials assessing the efficacy of interventions related to neurodegenerative diseases. A significant example is the serum urate concentration, which can be easily determined to identify gout, which is characterized by excess urate in the plasma and the deposition of solid uric acid in the joints. A randomized clinical trial (PRECEPT study) finalized several years ago with up to 800 patients with early PD identified serum urate concentration as the first biochemical parameter linked to the progression of “typical” PD (typical was the word used by the authors of the study) [71]. Shortly afterward and within the “Deprenyl and Tocopherol Antioxidative Therapy of Parkinsonism (DATATOP)” trial, in which more than 700 PD patients were enrolled, it was reported that slower rates of clinical decline were correlated with higher CSF and serum urate concentrations at baseline [72]. A later study with a smaller number of patients and different researchers/clinicians found data that supported serum urate concentration as a biomarker of PD [73]. One may wonder why this parameter is not included in the primary outcome measures.

PET imaging with tracers aimed at detecting amyloid plaques, or pTau, is promising and may serve to complete and refine diagnosis, but it cannot be used to measure neuroprotection (see Section 5). One concern is the difficulty in reaching an interpretative consensus. For example, clinical trial NCT03901105 requires flortaucipir scans to be verbatim “read by five independent, blinded readers”. Additionally, currently, PET scans for AD do not necessarily confirm the diagnosis on their own, as some individuals may have amyloid plaques without presenting symptoms of AD.

Neurometabolomics was a word coined in 2014 to highlight that determining the concentration in plasma or CSF of small molecules specifically related to brain metabolism could help “uncover potential biomarkers of aging and neurodegenerative diseases” [74]. This prediction is now more possible than ever due to recent methodological advancements that make it possible to simultaneously determine the concentration of hundreds of small compounds with a few microliters of sample. It should be noted that in a pioneering study, the metabolomics profile of CSF samples from AD patients and cognitively normal controls suggested that some of them had a high level of discrimination power to distinguish patients from controls [75]. To our knowledge, there has been no further validation of the selected compounds as biomarkers of AD. Accordingly, it has not been possible to assess whether the level of these differentially concentrated metabolites varies with disease progression. Hence, their potential as biomarkers of neuroprotection is not known.

**Figure 1:**
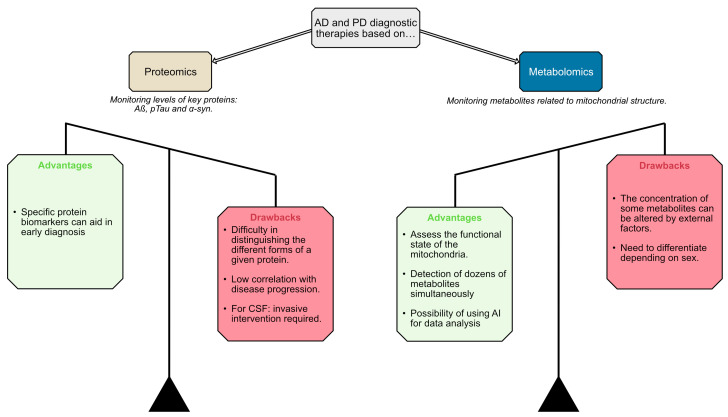
Pros and cons of proteomics and metabolomics for AD/PD diagnostics and therapeutics. Proteomics allows the detection of potentially useful biomarkers, but in clinical practice, it would be difficult to differentiate among the different versions (phosphorylated, glycosylated, etc.) of a given protein. Metabolomics may simultaneously detect hundreds of metabolites, including those that reflect mitochondrial dysfunction, but the combination of various metabolite levels may be necessary to achieve the right information on disease progression and/or the effectiveness of presumably neuroprotective interventions.

The metabolomics approach seems to offer more advantages over proteomics for AD and PD diagnostic and therapeutic development. The ability of metabolomics to concurrently assess mitochondrial functional state and detect dozens of metabolites, coupled with the potential for leveraging artificial intelligence (AI) in data analysis, appears more promising than proteomics. While metabolomics faces drawbacks such as the influence of external factors on metabolite concentrations and the lack of standardized methods, these challenges seem less severe compared to the difficulties proteomics encounters in distinguishing different protein forms and its low correlation with disease progression. Therefore, the metabolomics strategy may hold greater potential for facilitating early diagnosis and developing effective therapies for AD and PD (Figure 1). However, a combined application of both proteomics and metabolomics could potentially be beneficial for early disease diagnosis.

Pioneering studies in 2012 [76] and 2013 [77] used saliva to identify abnormal levels of small molecules in dementia patients. The first study found taurine useful for distinguishing between the saliva of controls and individuals diagnosed with mild cognitive impairment [76]. The second study reported significant differences in the concentrations of arginine and tyrosine between controls and dementia patients [77]. More recently, a pilot study using NMR-based metabolomics with saliva samples from healthy controls and individuals diagnosed with mild cognitive impairment or AD identified 22 metabolites with differential concentrations in the patients’ saliva [77]. In human CSF samples, the level of the SM(d18:1/18:0) sphingolipid increases in parallel to the level of pathological AD markers, tau, ptau-181, and Aß_42_ [78]. Metabolomics is serum from the Alzheimer’s Disease Neuroimaging Initiative that included controls and patients with cognitive impairment and AD, showed that “Metabolomics identified key disease-related metabolic changes and disease-progression-related changes” [79]. Also in 2017, 12 metabolites were identified with concentrations in plasma inversely correlated when comparing older adults with superior memory and patients with mild cognitive impairment from prodromic AD [80]. In 2015, the same laboratory identified a panel of 24 metabolites, 22 of which were dysregulated lipids in plasma. These metabolites demonstrated positive and negative predictive values in predicting progression from mild cognitive impairment to AD [81]. It is puzzling why, seven years after these studies, salivary or plasma/serum parameters are still not considered in AD-related clinical trials listed in Table 4. The technical burden associated with pioneering studies has been surpassed by new technological developments, leading to a quantum leap in the number of metabolites that can be identified and quantified in a given sample. In fact, these studies used a novel technological platform (“Biocrates”, see below).

Metabolomic studies have already been performed on several transgenic animal models of AD. The results show alterations in the urea cycle, the Krebs cycle, the intermediate metabolism, and the homeostasis of amino acids and lipid metabolism (reviewed in [82,83]). It is becoming more and more evident that mitochondrial dysfunction is a hallmark of both PD and AD, and these mitochondrial alterations may manifest as differentially concentrated metabolites in plasma and CSF [83,84]. The careful selection of key metabolites related to mitochondrial metabolism exhibiting distinct profiles in PD and AD could enable an unambiguous differential diagnosis. 

The field has rapidly gained high confidence in Biocrates technology, which has advanced to enable the simultaneous measurement of up to 500 small metabolites from just a few microliters of body fluid samples (source: https://biocrates.com/; accessed on 17 July 2024). We utilized this innovative technology to analyze approximately 35 µL of aqueous humor from control subjects, type 2 diabetes patients, and PD patients. The results identified 3–5 metabolites with molecular weights below 1000 Da, enabling highly sensitive and specific disease diagnosis [85]. This approach leverages novel analytical techniques capable of detecting and quantifying a wide range of metabolites, including amino acids, lipids, and acylcarnitines. Importantly, this metabolomic strategy is readily translatable to other biofluids such as serum, urine, or CSF, and could be implemented within AI systems, potentially becoming a powerful tool for personalized and precision medicine in the coming years. The discovery of biomarkers of diagnosis and the approaches followed to detect them will be instrumental in discovering biomarkers of neuroprotection. It is tempting to speculate that not all the small molecules that become biomarkers of the diagnosis of neurodegenerative disease will be biomarkers of neuroprotection and that not all small-molecule biomarkers of neuroprotection are altered in patients with neurodegeneration.

**Table 4:** Clinical trials related to Alzheimer’s disease. Selected as being registered on Clinicaltrials.org, and for being interventional and having results. See details in text. Characteristics: 65 years or older; phase 3, interventional with results.

Ref Number	Title	Primary Outcome Measures	Biomarkers	Country and Year of Completion
NCT04623242	Dominantly Inherited Alzheimer Network Trial: An Opportunity to Prevent Dementia. A Study of Potential Disease Modifying Treatments in Individuals at Risk for or with a Type of Early Onset Alzheimer’s Disease Caused by a Genetic Mutation.	Assess Cognitive Efficacy in Individuals with Mutations Causing Dominantly Inherited AD as Measured by the DIAN-Multivariate Cognitive Endpoint (DIAN-MCE);	No	US 2019
NCT01224106	A Study of Gantenerumab in Participants with Prodromal Alzheimer’s Disease	1 Mean Change from Baseline in Clinical Dementia Rating Scale Sum of Boxes (CDR-SOB) Total Score at Week 104 (Double-Blind Treatment Phase) 2 Number of Participants with Adverse Events or Serious Adverse Events (OLE Phase)	No	US 2020
NCT02051608	A Study of Gantenerumab in Participants with Mild Alzheimer Disease	1 Part 2: Percentage of Participants with Adverse Events (AEs) or Serious Adverse Events (SAEs) 2 Part 2: Percentage of Participants with Treatment-Emergent Anti-Drug Antibodies (ADAs) 3 Part 2: Percentage of Participants with Adverse Events Leading to Discontinuation of Treatment	No	US 2021
NCT02484547	221AD302 Phase 3 Study of Aducanumab (BIIB037) in Early Alzheimer’s Disease	Change from Baseline in Clinical Dementia Rating Scale—Sum of Boxes (CDR-SB) Score at Week 78	No	US 2019
NCT02477800	221AD301 Phase 3 Study of Aducanumab (BIIB037) in Early Alzheimer’s Disease	Change from Baseline in Clinical Dementia Rating Sum of Boxes (CDR-SB) Score at Week 78	No	US 2019
NCT03114657	A Study of Crenezumab Versus Placebo to Evaluate the Efficacy and Safety in Participants with Prodromal to Mild Alzheimer’s Disease (AD)	Change from Baseline to Week 77 in Clinical Dementia Rating-Sum of Boxes (CDR-SB) Scale Score	No	US 2019
NCT02670083	A Study Evaluating the Efficacy and Safety of Crenezumab Versus Placebo in Participants with Prodromal to Mild Alzheimer’s Disease (AD).	Change from Baseline to Week 105 in Clinical Dementia Rating-Sum of Boxes (CDR-SB) Score	No	US 2019
NCT03491150	An Open-Label Crenezumab Study in Participants with Alzheimer’s Disease	1 Percentage of Participants with Adverse Events (AEs) and Serious Adverse Events (SAEs) 2 Percentage of Participants with Anti-Crenezumab Antibodies	No	US 2019
NCT05108922	A Study of Donanemab (LY3002813) Compared with Aducanumab in Participants With Early Symptomatic Alzheimer’s Disease (TRAILBLAZER-ALZ 4)	1 Percentage of Participants Who Reach Complete Amyloid Plaque Clearance on Florbetapir F18 Positron Emission Tomography (PET) Scan (Superiority) on Donanemab Versus Aducanumab 2 Percentage of Participants Who Reach Complete Amyloid Plaque Clearance on Florbetapir F18 PET Scan in the Low/Medium (Intermediate) Subpopulation (Superiority) on Donanemab Versus Aducanumab	PET *	US 2023
NCT03131453	A Study of CNP520 Versus Placebo in Participants at Risk for the Onset of Clinical Symptoms of Alzheimer’s Disease	1 Time to Event (Diagnosis of Mild Cognitive Impairment or Dementia, Due to Alzheimer’s Disease (AD)) 2 Change in the Alzheimer’s Prevention Initiative Composite Cognitive (APCC) Test Score	No	US 2020
NCT02565511	A Study of CAD106 and CNP520 Versus Placebo in Participants at Risk for the Onset of Clinical Symptoms of Alzheimer’s Disease	1 Time to Event (Diagnosis of Mild Cognitive Impairment or Dementia, Due to Alzheimer’s Disease (AD)) 2 Change in the Alzheimer’s Prevention Initiative Composite Cognitive (APCC) Test Score	No	US 2020
NCT02442765	Efficacy, Safety, and Tolerability of AVP-786 for the Treatment of Agitation in Patients with Dementia of Alzheimer’s Type	Stage 1 and Stage 2: Change from Baseline in the Cohen-Mansfield Agitation Inventory (CMAI) Composite Score to Week 6 and Week 12	No	US 2019
NCT02442778	Efficacy, Safety, and Tolerability of AVP-786 for the Treatment of Agitation in Participants with Dementia of Alzheimer’s Type	Change from Baseline to Week 12 in the Cohen-Mansfield Agitation Inventory (CMAI) Composite Score	No	US 2019
NCT03548584	A Trial to Evaluate the Safety, Efficacy, and Tolerability of Brexpiprazole in Treating Agitation Associated with Dementia of the Alzheimer’s Type	Change from Baseline to Week 12 in the CMAI Total Score	No	US 2022
NCT03226522	Addressing Dementia Via Agitation-Centered Evaluation	(efficacy and safety of AXS-05) Change in CMAI Total Score. Cohen-Mansfield Agitation Inventory (CMAI) is a 29-item caregiver-rated questionnaire that assesses the frequency of agitation-related and disruptive behaviors in subjects with dementia.	No	US 2020
NCT03721705	Renew NCP-5 for the Treatment of Mild Cognitive Impairment Due to Alzheimer’s Disease (AD) or Mild Dementia of Alzheimer’s Type	Mean Change from Baseline to 24 Weeks in Vascular Dementia Assessment Scale Cognitive Subscale (vADAS-cog) Using the Average of Scores at 12, 18 and 24 Weeks.	No	US 2021
NCT02956486	A 24-Month Study to Evaluate the Efficacy and Safety of Elenbecestat (E2609) in Participants with Early Alzheimer’s Disease	Mean Change from Baseline to 24 Weeks in Vascular Dementia Assessment Scale Cognitive Subscale (vADAS-cog) Using the Average of Scores at 12, 18 and 24 Weeks.	No	US 2021
NCT02956486	A 24-Month Study to Evaluate the Efficacy and Safety of Elenbecestat (E2609) in Participants with Early Alzheimer’s Disease	1 Core Phase: Change from Baseline up to Month 24 in the Clinical Dementia Rating-sum of Boxes (CDR-SB) Score 2 Extension Phase: Number of Participants Reporting One or More Treatment-emergent (serious and non-serious) Adverse Events (TEAEs)	No	US 2020
NCT02972658	A Study of Lanabecestat (LY3314814) in Early Alzheimer’s Disease Dementia	Change from Baseline Analysis on the 13-item Alzheimer’s Disease Assessment Scale—Cognitive Subscale (ADAS-Cog13)	No	US 2018
NCT02916056	2-Year Extension Study of Azeliragon in Subjects with Alzheimer’s Disease (STEADFAST Extension)	Number of Subjects with at Least One Treatment-Emergent Adverse Event	No	US 2018
NCT02586909	12-Month Open-Label Extension Study of Intepirdine (RVT-101) in Subjects with Alzheimer’s Disease: MINDSET Extension	Occurrence of Adverse Events (AEs) and or Reported Changes in Physical Examinations, Vital Signs Measurements, Electrocardiograms (ECGs), Routine Laboratory Assessments	No	US 2018
NCT02783573	A Study of Lanabecestat (LY3314814) in Participants with Mild Alzheimer’s Disease Dementia	Change from Baseline in Alzheimer’s Disease Assessment Scale- Cognitive Subscale (ADAS-Cog13) Score	No	US 2018
NCT02350127	Preventing Loss of Independence Through Exercise (PLIE) in Persons with Dementia	1 Quality of Life Scale in Alzheimer’s Disease (QOL-AD) 2 Short Physical Performance Battery (SPPB)-Modified 3 Alzheimer’s Disease Assessment Scale—Cognitive Subscale (ADAS-cog)	No	US 2018
NCT02080364	Evaluation of the Efficacy and Safety of Azeliragon (TTP488) in Patients with Mild Alzheimer’s Disease	1 Change from Baseline in (ADAS-cog) Total Score 2 Change from Baseline in Clinical Dementia Rating Scale Sum of Boxes (CDR-sb)	No	US 2018
NCT01561053	A Study to Evaluate Albumin and Immunoglobulin in Alzheimer’s Disease	1 Alzheimer’s Disease Assessment Scale—Cognitive Subscale (ADAS-Cog) Total Score (Changes from Baseline to 14 Months) 2 Alzheimer’s Disease Cooperative Study-Activities of Daily Living (ADCS-ADL) Total Score (Changes from Baseline to 14 Months)	No	US 2018
NCT03823404	GAIN Trial: Phase 2/3 Study of COR388 in Subjects with Alzheimer’s Disease	1 Alzheimer’s Disease Assessment Scale-Cognitive Subscale 11 (ADAS-Cog 11) 2 Alzheimer’s Disease Cooperative Study Group-Activities of Daily Living (ADCS-ADL)	No	US 2022
NCT03901105	Evaluation of Flortaucipir PET Signal and Cognitive Change in Early Alzheimer’s Disease	Risk Ratio for AD Symptom Progression on Clinical Dementia Rating Scale Sum of Boxes (CDR-SB) scale	PET *	US 2022
NCT03901092	A Reader Study to Assess Accuracy and Reliability of Flortaucipir F 18 Positron Emission Tomography (PET) Scan Interpretation	1 Primary Objective 1 Analysis 1: Diagnostic Performance of Individual Readers (NFT Score) 2 Primary Objective 1 Analysis 2: Diagnostic Performance of Individual Readers (NIA-AA Autopsy Diagnosis) 3 Primary Objective 2: Inter-reader Reliability of Reader Interpretation of Flortaucipir-PET Imaging	PET *	US 2019
NCT02516046	18F-AV-1451 Autopsy Study	1 Primary Outcome 1: Diagnostic Performance of Individual Readers (NFT Score) 2 Primary Outcome 2: Diagnostic Performance of Individual Readers (NIA-AA Autopsy Diagnosis)	PET *	US 2018
NCT01953601	Efficacy and Safety Trial of Verubecestat (MK-8931) in Participants with Prodromal Alzheimer’s Disease (MK-8931-019)	1 Part 1 (Base Study). Least Squares Mean (LSM) Change from Baseline in Clinical Dementia Rating Sum of Boxes (CDR-SB) Score at Week 104 2 Part 2 (Extension Study). Mean Change from Baseline in Clinical Dementia Rating Sum of Boxes (CDR-SB) Score at Week 130 3 Part 1 (Base Study). Percentage of Participants Who Experienced ≥ 1 Adverse Event (AE) 4 Part 1 (Base Study). Percentage of Participants Who Discontinued from Study Drug Due to an Adverse Event (AE) 5 Part 2 (Extension Study). Percentage of Participants Who Experienced ≥ 1 Adverse Event (AE) 6 Part 2 (Extension Study). Percentage of Participants Who Discontinued from Study Drug Due to an Adverse Event (AE)	No	Not provided 2018
NCT02346201	Apathy in Dementia Methylphenidate Trial 2	1 Neuropsychiatric Inventory (NPI) 2 Percentage of Participants with Change in Modified Alzheimer’s Disease Cooperative Study- Clinical Global Impression of Change (CGIC)	No	US 2020
NCT02245737	An Efficacy and Safety Study of Lanabecestat (LY3314814) in Early Alzheimer’s Disease	Change from Baseline on the 13-item Alzheimer’s Disease Assessment Scale—Cognitive Subscale (ADAS-Cog13)	No	US 2018
NCT02817906	ITI-007 for the Treatment of Agitation in Patients with Dementia, Including Alzheimer’s Disease	Change from Baseline in the Cohen-Mansfield Agitation Inventory—Community Version (CMAI-C)	No	US 2019
NCT01767909	The Study of Nasal Insulin in the Fight Against Forgetfulness (SNIFF)	Change in Global Measure of Cognition as Measured by the Alzheimer’s Disease Assessment Scale-Cognitive 12 (ADAS-Cog12)	No	US 2018
NCT02284906	AD-4833/TOMM40_303 Extension Study of the Safety and Efficacy of Pioglitazone to Slow Cognitive Decline in Participants with Mild Cognitive Impairment Due to Alzheimer’s Disease	Change from Extension Study Baseline in Composite Score of a Broad Cognitive Test Battery at Month 24	No	US 2018
NCT01931566	Biomarker Qualification for Risk of Mild Cognitive Impairment (MCI) Due to Alzheimer’s Disease (AD) and Safety and Efficacy Evaluation of Pioglitazone in Delaying Its Onset	1 Time to Diagnosis of Mild Cognitive Impairment Due to Alzheimer’s Disease (MCI-AD) for Placebo-treated, High-risk, Non-Hispanic/Latino Caucasian Participants Versus Placebo-treated, Low-risk, Non-Hispanic/Latino Caucasian Participants 2 Time to Diagnosis of MCI Due to AD for Pioglitazone-treated, High-risk, Non-Hispanic/Latino Caucasian Participants Versus Placebo-treated, High-risk, Non-Hispanic/Latino, Caucasian Participants	No	US 2018
NCT02569398	An Efficacy and Safety Study of Atabecestat in Participants Who Are Asymptomatic at Risk for Developing Alzheimer’s Dementia	Change from Baseline in Preclinical Alzheimer Cognitive Composite (PACC) Score at Endpoint (Month 24)	No	US 2018
NCT02750306	Safety and Efficacy of Suvorexant (MK-4305) for the Treatment of Insomnia in Participants with Alzheimer’s Disease (MK-4305-061)	1 Change from Baseline in Polysomnography-derived Total Sleep Time (TST) at Week 4 2 Percentage of Participants Who Experienced One or More Adverse Events 3 Percentage of Participants Who Discontinued Study Drug Due to an Adverse Event	No	US 2018
NCT02248636	Cholinesterase Inhibitor Discontinuation	Successful Completion	No	US 2019
NCT03325556	Relapse Prevention Study of Pimavanserin in Dementia-related Psychosis	Time from Randomization to Relapse in the Double-blind (DB) Period	No	US 2019

* PET. Positron emission tomography can be used as a surrogate for amyloid burden. There is no consensus on how to interpret the data to reliably measure the degree of improvement with any AD intervention.

In conclusion, the development of disease-modifying therapies for neurodegenerative conditions like PD and AD has been hindered by the lack of reliable biomarkers to accurately assess neuroprotective effects. While current clinical trials predominantly rely on cognitive/motor test scores and neuroimaging techniques like PET, these measures have inherent limitations and do not directly diagnose. The novel metabolomic approaches discussed here, focused on detecting mitochondria-related metabolite signatures in biofluids, offer a promising alternative for developing robust biomarkers of disease progression and neuroprotection. The vast amount of data that can now be generated requires specific tools for proper analysis and biomarker discovery. The use of bioinformatics, data mining, and statistically relevant discriminant tools is thus essential [82,86,87,88]. It is also relevant to notice that one possibility that now arises is the combination of various parameters, i.e., the concentration of various metabolites, to assess diagnosis and/or disease progression [85].

In addition to providing critical insights into the metabolic changes in AD and PD, metabolomics must complement proteomics in the search for biomarkers in plasma and CSF. Analyzing metabolites in urine and saliva offers non-invasive methods to identify biomarkers for early diagnosis and disease monitoring in AD and PD. In fact, the study of metabolomics in plasma, CSF, urine, and saliva can uncover unique metabolic signatures associated with AD and PD, aiding in the development of targeted treatments. Including multiple biological specimens in metabolomic studies broadens our understanding of the biochemical alterations in AD and PD, improving diagnostic and therapeutic strategies.

## 8. Conclusions

There is currently no marker that can be used to assess neuroprotection in clinical trials. Clinical trials related to Parkinson’s and Alzheimer’s diseases do not use any criteria to evaluate neuroprotection. The field of biomarker discovery has been very active in looking for molecules, in animal models and patients, that are markers of neuronal death and/or disease progression. Efforts have focused on measuring, in plasma and/or CSF, the level of proteins that aggregate in these diseases. Limitations of the proteomics approach include uncertainties on which proteins must be determined: monomer, dimer, phosphorylated, etc., and whether there is a correlation between a decrease in protein concentration and real neuroprotection (Figure 1).

Advances in technology to measure hundreds of molecules in a few microliters of sample allow us to consider metabolomics as a promising approach to detecting metabolic alterations in patients and their correlations with neurodegeneration. Being able to measure several parameters with the same sample and in longitudinal studies looks promising for detecting selective PD or selective AD biomarkers. Relevant is the possibility of using discriminant analysis to select a combination of 3–4 parameters, each being the concentration of a molecule, to make the diagnosis, the assessment of the advance of the disease, and/or the assessment of the neuroprotective efficacy of a novel therapy. The main limitation, which is transversal to all approaches, is the need to select first biomarkers that correlate with the disease, i.e., biomarkers that could be used for diagnosis. Steps are needed to go forward and discover biomarkers of neuroprotection. Again, it is important to note that several parameters may be useful for diagnosis and assessing neuroprotection. In other words, the approach is robust at both stages—diagnosis and assessment of disease progression—because the concentration of some of the metabolites may be appropriate for diagnosis and not for assessing neuroprotection, and vice versa. Consequently, the metabolomic approach should be validated first for diagnosis and then used to evaluate disease progression/neuroprotection.

Proteomics and metabolomics should be used together, as they can provide complementary insights. This synergy has been demonstrated in studies using tears, a readily accessible yet often undervalued body fluid (see [89] for review). Looking toward the future, it is crucial to integrate advanced metabolomic techniques and previous metabolomic-based findings (even using body fluids from patients) into larger-scale clinical trials to validate their efficacy in real-world settings. 

Emerging technologies, such as artificial intelligence and machine learning, could play a significant role in analyzing complex metabolomic data to identify novel biomarkers and predict disease trajectories. In this regard, a recent study is considered pioneering for reporting “an interpretable neural network (NN) framework to accurately predict disease and identify significant biomarkers using whole metabolomics data sets without a priori feature selection” [90]. The authors are confident that their approach may be useful for diagnosing several diseases by integrating metabolomics data with other untargeted “omics” data. Furthermore, interdisciplinary collaborations between clinicians, researchers, and data scientists will be essential to developing robust, reproducible, and clinically relevant biomarkers. As our understanding of neurodegenerative diseases evolves, continuous investment in biomarker research will pave the way for more effective therapeutic strategies and personalized medicine approaches, ultimately improving patient outcomes and quality of life.

## Figures and Tables

**Table 1:** Recent results from neuroprotective interventions in animal models of Parkinson’s disease.

Intervention	Effect	Mechanism	Model	Reference
Kurarinone (from *Sophora flavescens*)	Attenuating the 1-methyl-4-phenyl-1,2,3,6-metrahydropyridine (MPTP)-mediated neuroinflammation.	Suppress proinflammatory activation of microglia via the nuclear factor kappa B signaling pathway. (Having the soluble epoxide hydrolase (sEH) as a promising target).	MPTP-induced PD mice.	[16]
Tauroursodeoxycholic acid (TUDCA)	Anti-apoptotic and neuroprotective activity, and acts as a chemical chaperone to maintain the stability and correct folding of proteins.	Inhibition of the intrinsic mitochondrial apoptotic pathway, through the reduction of ROS and inactivation of BAX. Probable decrease in the expression of genes involved in cell cycle regulation (Cyclin D1).	Transgenic mice and *Caenorhabditis elegans*.	[17]
Celastrol	Protects against dopaminergic neuron loss, mitigates neuroinflammation, and alleviation of motor deficits.	Unknown.	MPTP-induced PD mice and AAV-mediated human α-synuclein overexpression	[18]
Curcumin	Neuroprotective effects, reduction of motor deficits, and decreased neuroinflammation.	Gut-brain axis. Modification of the levels of key amino acids.	MPTP-induced PD mice	[19]
Urolithin A	Improvement of motor deficits and dopaminergic neurodegeneration, promotion of autophagy and mitophagy, and reduction of neuroinflammation.	Reduced expression of pro-inflammatory factors including IL-1ß, TNF-α, or iNOS. Reducing NLRP3 inflammasome activation.	MPTP-induced PD mice	[20]
Osmotin	Reduces neuronal damage, improves motor function, and decreases the accumulation of alpha-synuclein aggregates.	Multiple pathways affected: AdipoR1 Pathway, MAPK Pathway, AMPK Pathway, and mTOR.	Rat PD models (6-hydroxydopa mine or rotenone)	[21]
Withaferin A	Protects against loss of dopaminergic neurons, neuroinflammation, and motor deficits. Also alleviates accumulation of phosphorylated α-synuclein.	Unknown.	MPTP-induced PD mice	[22]
Ceftriaxone	Alleviates the behavioral and neuropathological changes induced by MPTP. (neuroprotective effect)	Reducing the expression of neuroinflammation-related Toll-like receptor 4 (TLR4), and the phosphorylated nuclear factor kappa-B in the brain of PD mice.	MPTP-induced PD mice	[23]
Dl-3-n-Butylphthalide	Attenuates neuroinflammation and the aggregation of α-Syn. Alleviating neuroinflammation and reducing mitochondrial function impairment.	Impairment of the activation of the NLRP3 inflammasome and of PARP1.	MPTP-induced PD mice	[24]
Necrosulfonamide	Inhibiting cell death, alleviating, neuroinflammation, and reducing α-synuclein oligomerization.	Inhibition of the mixed lineage kinase domain-like protein (MLKL), which mediates necroptosis. Inhibition of proinflammatory activation of microglia activation and reactive astrogliosis. Reduction in the expression of proinflammatory factors such as tumor necrosis factor-α and interleukin-1ß.	MPTP-induced PD mice	[25]

**Table 2:** Recent results from neuroprotective interventions in animal models of Alzheimer’s disease.

Intervention	Effect	Mechanism	Model	Reference
N-[3-([1,1-Biphenyl]-4-yloxy)-3-(4-fluorophenyl)propyl]-N-methylglycine (NFPS)	Cognitive improvement in short-term and long-term memory, novel object recognition, and spatial memory tasks.	GlyT1 inhibition	Intra-hippocampal injection of amyloid-ß to mice	[26]
Norboldine	Improvement in the capability of learning and reducing Aß deposition.	AMPK/GSK3ß/Nrf2 pathway.	3 × Tg mice	[27]
Chlorogenic Acid (CGA)	Neuroprotective effects.	Decreased oxidative stress and reduced neuroinflammation.	Intracerebroventricular administration of streptozotocin	[28]
Butyrylcholinesterase inhibitor UW-MD-95	Prevention of Aß_25-35_-induced oxidative stress (assessed by lipid peroxidation or cytochrome c release), neuroinflammation (IL-6 and TNFα levels or GFAP and IBA1 immunoreactivity) in the hippocampus and cortex, and apoptosis (Bax level). Reversing the increase in soluble Aß_1-42_ levels in the hippocampus.	Butyrylcholinesterase (BChE), affects acetylcholine turnover and is likely involved in the formation of Aß aggregates.	Aß_25-35_-induced mice model	[29]
RG2833	Mitigation of hippocampal-dependent spatial memory deficits.	Modulating the expression of immediate early, neuroprotective, and synaptic plasticity genes.	TgF344-AD rats	[30]
Gas-miR36-5p	Neuroprotective properties	Targeting GSK-3, which participates in tau hyperphosphorylation.	AD cell model	[31]
TCR_Aß_-Tregs	Reduction of amyloid burden and reversing cognitive deficits.	TCR_Aß_-Tregs, which target amyloid-rich regions in the brain and lead to neuroprotective outcomes.	APP/PS1 mice	[32]
Exercise training and postbiotic supplement	Reduction of amyloid burden Reduction of mutant APP gene expression	NF-kB signaling pathway	APP/PS1 mice	[33]
Isolinderalactone	Reversing learning and memory deficits. Reduction of Aß-plaque deposition and neuronal death.	JNK signaling pathway Impaired synaptic plasticity and glial cell activation.	APP/PS1 mice	[34]
N-methyl-(2S, 4R)-trans-4-hydroxy-L-proline (NMP)	Reversing the alterations related to synaptic contacts. Reversing cognitive deficits.	In part via inhibiting activaneuroinflammation	Aß_1-42_-injected mouse model	[35]
Aucubin	Improved the behaviors, counteracted cognitive and memory deficits, and ameliorated deposition of Aß plaques, neuronal damage, and inflammatory responses induced by glial cell overactivation.	Inhibition of ERK-FOS axis.	APP/PS1 mice	[36]
Methionine	Lower dietary methionine intake is associated with improved cognitive function. Restored synapse ultrastructure and alleviated mitochondrial dysfunction. Balanced the redox status and activated cystathionine-ß-synthase (CBS)/H_2_S pathway in the brain.	CBS/H_2_S pathway plays an essential role. Also, enhanced mitochondrial biogenesis in the brain.	APP/PS1 mice	[37]
Conifer Essential Oils	Attenuated memory impairments, with *P. halepensis* Reduced IL-1ß expression and induced positive effects against DNA fragmentation.	Modulating BDNF and ARC Expression	Aß_1-42_-injected rat model	[38]
TO901317	Alleviates the impairment of memory, decreases Aß aggregates, and increases proteasome activity.	These effects were blocked by cotreatment with an LXR antagonist (GSK2033).	APP/PS1 mice	[39]
Anthocyanin-rich blueberry extracts and anthocyanin metabolite protocatechuic acid	Neuron damage in morphology was reduced and the expression of autophagy-related proteins was promoted.	Mechanism of BBE for reducing neuronal damage by promoting neuronal autophagy.	APP/PS1 mice	[40]
Geniposidic Acid	Improved cognitive impairment, reducing Aß accumulation and neuronal apoptosis. Alleviated inflammation and axonal injury of Aß_1-42_-induced neurons.	GAP43 was shown experimentally to be the target of GPA in AD. Silencing of GAP43 repressed the neuroprotective effect of GPA. GPA elevated GAP43 expression via PI3K/AKT pathway activation and ultimately improved nerve injury.	mPrP-APPswe/PS1De9 mice	[41]
JOTROL (resveratrol formulation)	Increased bioavailability of resveratrol. Modulation of the expression of AD-related genes (Adam10, Bace1, Bdnf, Psen1).	Increased expression of neuroprotective genes, and suppression of pro-inflammatory genes.	3xTg-AD	[42]
Nano-Honokiol	Prevents tau hyperphosphorylation. Suppressed neuroinflammatory response. Regulated composition of gut microbiota.	Inhibiting neuropathology and modulating gut microbiota. Modulating JNK/CDK5/GSK-3Beta pathway. Inhibited activation of microglia, astrocyte, and Abeta burdens.	TgCRND8 mice	[43]
